# Development of a Breech-Specific Integrated Care Pathway for Pregnant Women: Protocol for a Mixed Methods Study

**DOI:** 10.2196/23514

**Published:** 2021-02-23

**Authors:** Sara Morris, Sadie Geraghty, Deborah Sundin

**Affiliations:** 1 King Edward Memorial Hospital Subiaco Australia; 2 Edith Cowan University Joondalup Australia

**Keywords:** breech presentation, midwifery, methodology, Delphi technique

## Abstract

**Background:**

The development of an integrated care pathway with multidisciplinary input to standardize and streamline care for pregnant women experiencing breech presentation at 36 or more weeks of gestation poses several challenges because of the divisive and contentious nature of the phenomenon. Although many clinicians are interested in obtaining the skills required to safely support women desiring a vaginal breech birth, the primary trend in most health care facilities is to recommend a cesarean section.

**Objective:**

This paper aims to discuss the mixed methods approach used in a doctoral study conducted to generate new knowledge regarding women’s experiences of breech birth in Western Australia and professional recommendations regarding the care of women experiencing breech presentation close to or at term. This study was designed to inform the development of an integrated care pathway for women experiencing a breech presentation. This mixed methods approach situated within the pragmatic paradigm was determined to be the optimal way for incorporating multidisciplinary recommendations with current clinical practice guidelines and consumer feedback.

**Methods:**

A mixed methods study utilizing semistructured interviews, an electronic Delphi (e-Delphi) study, and clinical practice guideline appraisal was conducted to generate new data. The interviews were designed to provide insights and understanding of the experiences of women in Western Australia who are diagnosed with a breech presentation. The e-Delphi study explored childbirth professionals’ knowledge, opinions, and recommendations for the care of women experiencing breech presentation close to or at term. The clinical practice guideline appraisal will examine the current national and professional breech management and care guidelines. This study has the potential to highlight areas in practice that may need improvement and enable clinicians to better support women through what can be a difficult time.

**Results:**

Data collection for this study began in November 2018 and concluded in March 2020. Data analysis is currently taking place, and the results will be disseminated through publication when the analysis is complete.

**Conclusions:**

The results of this study will guide the development of an integrated care pathway for women experiencing a breech presentation close to or at term, with the hope of moving toward standardized breech care for women in Western Australia. This study protocol has the potential to be used as a research framework for future studies of a similar nature.

**International Registered Report Identifier (IRRID):**

DERR1-10.2196/23514

## Introduction

### Background

Birth of any kind, vaginal or cesarean section (CS), is not without risk [1]. The safest mode of birth for women experiencing breech presentation has created debate among academics and practicing clinicians for over 20 years [2]. Women who experience breech presentation late in the third trimester (between 35 and 42 weeks) are usually recommended to have a CS [2,3]. The *Term Breech Trial* [3] had a significant effect on breech birth practices, suggesting that CS was the safest mode of birth for women experiencing breech presentation despite concerns raised regarding the validity of the study [1,4,5]. A subsequent study by the same research team revealed no significant differences in the developmental outcomes of breech born children at 2 years of age, regardless of their birth mode [6]. However, this did not reverse the dramatic rise of the CS rate for breech presentation, which ranges from 69% to 100% [2]. The persistence of this trend has led to the deskilling of practitioners in relation to vaginal breech birth (VBB) [7] and a subsequent limitation of birth choices for women in most settings. The implications for women who have a CS include an increased risk of infection and deep vein thrombosis, greater postpartum blood loss, longer hospital stays, risk of complications such as placenta accreta, increta, and percreta (abnormal implantation of the placenta), and uterine rupture in subsequent pregnancies [8-11]. There is also evidence suggesting that the birth mode has the potential to affect the long-term health outcomes of children. Owing to a lack of exposure to their maternal microbiome, cesarean birth puts children at risk of metabolic diseases such as diabetes and respiratory issues such as asthma [11,12]. Despite leading guidelines supporting balanced, unbiased counseling and the option of a VBB for women meeting eligibility criteria [13], VBB is still not offered in many settings. However, VBB continues to occur and women still seek out clinicians who are supportive of their decision to attempt a vaginal birth [2]. Women have reported receiving limited information, the use of biased and coercive counseling techniques from clinicians to dissuade them from attempting a VBB, and a perceived lack of support from within their social support network (ie, family and friends) in their desire for a VBB, suggesting that negative views of VBB exist in the wider society [14,15]. Women in these studies valued balanced, evidence-based information and nonjudgmental counseling from clinicians who are experienced in and supportive of VBB [14,15].

Women come to decisions about birth mode through a complex interplay among their own preferences, social influences, clinicians’ views and experiences, and health system support [16]. Diagnosis of a breech presentation in late pregnancy has been reported as a stressful event, as it was seen to change the trajectory of the pregnancy journey from one of relative normalcy to one of risk [15]. Previous Australian research exploring women’s knowledge of breech presentation management and the mode of birth preferences regardless of presentation at birth indicated that 90.8% (158/174) of women favored a vaginal birth over a CS [17]. This has been supported by a research from the Netherlands that indicated that approximately 40% of women experiencing breech presentation at term wanted the opportunity for a vaginal birth, even if treatments such as an external cephalic version (ECV) failed [5].

Media representations of breech birth tend to emphasize the risks of a vaginal birth and focus on the relative safety of CS, which contributes to the societal perception of VBB as a dangerous option [18]. Women considering options that diverge from common practice (ie, ECV and CS) have reported the use of bullying and scare tactics when expressing their preference for a vaginal birth [15]. These women potentially face pressure from within the health care system and without. Australian-based breech studies have found that women with a breech fetus at term desiring a VBB often experience pressure from their families to have a CS [19].

However, with the growing international concern regarding the escalating rate of cesarean birth and its potential consequences for childbearing women and their current and possible future children [20], there has been a focus on health initiatives that aim at *normalizing* birth or reducing the rate of medical intervention [21]. Such initiatives include the implementation of specialized breech teams or clinics. Specialized breech teams or clinics and care pathways have been recommended as a way of providing balanced counseling and support for women during the decision-making process and, by extension, support their birth choices [22]. Specialized breech clinics also offer clinicians the opportunity to enhance and maintain their breech birth skill set, which is key to the safety of breech births [1,23,24]. Currently, although there are only 2 specialty breech clinics in Australia, both of which are in New South Wales (John Hunter Hospital and Women’s and Newborn Health Westmead Hospital) [25], the midwives and obstetricians in such clinics work collaboratively to provide women with information concerning procedures to promote a head-down fetal position (ie, cephalic version) and birth mode options [23]. They have also been shown to decrease the rate of CS for breech by improving the uptake and success of ECV [26,27]. Furthermore, they have had success through the encouragement of women deemed suitable to opt for or continue to a VBB where ECV has failed, is contraindicated, or has been declined [27].

An integrated care pathway (ICP) is a formalized document which outlines the ideal pathway of care for people experiencing a particular health phenomenon and has been broadly used across aspects of health care [28-31]. ICPs have been shown to reduce hospital-related complications and the length of stay and to improve clinical documentation and patient satisfaction [29,32]. They also reportedly promote patient-focused care, facilitate patient education regarding their health phenomena, facilitate collaboration within the multidisciplinary health team, and introduce evidence-based care and treatments available to patients that can be adapted to suit local conditions while reducing costs and maximizing resources (ie, by minimizing unnecessary tests or procedures) [28]. A midwifery ICP was developed to promote vaginal births by Clarke et al, after an internal review showed that routine midwifery-led care sometimes resulted in unnecessary interventions. The vaginal birth ICP described by Clarke et al [30] was seen to legitimize the midwifery model of care in the Wales context, promoting the midwifery role and redefining the midwifery territory, however it did not meet its original aim of reducing the CS rate [33,34]. Other ICPs used in perinatal care have been shown to improve communication between clinicians and consumers and increase consumer satisfaction [29]. In Australia, a range of clinical pathways for maternity care exist. These include a CS pathway, vaginal birth pathway, an assisted vaginal birth pathway, as well as various neonatal pathways and community care program pathways [31].

Database searches exposed reference to existing breech-specific ICPs; however, one could neither be found in the Australian context nor were the authors able to obtain a copy of an existing breech-specific ICP. An ICP for breech presentation has the potential to reduce unnecessary intervention and streamline care including timely referral and intervention and aid in promoting nonbiased counseling.

### Objectives

Owing to the potential benefits of ICP, the results of this study will be used to guide the development of an ICP for women diagnosed with a breech presentation close to or at term (ie, approximately 35-40 weeks of gestation) to support the delivery of high-quality, evidence-based care. The development of a breech-specific ICP for women in Western Australia will be achieved through the amalgamation of expert opinion (ie, consensus reached in the electronic Delphi [e-Delphi] study), consumer feedback (ie, based on results from interviews exploring women’s experiences of breech birth), and the incorporation of breech care guidelines [30].

## Methods

### Research Questions

The primary questions this research aimed to answer were the following:

- What barriers and facilitators do women experiencing breech presentation close to or at term experience in Western Australia?
- What optimal pathway of care is recommended for women with a breech presentation between 36 and 42 weeks?

### Pragmatism

In the context of research, a *paradigm* refers to the philosophical assumptions that direct the researcher and describe their worldview [35]. Pragmatism as a research paradigm proposes that the researchers use methodological and philosophical approaches that will work best to answer the problem in focus [35,36]. Pragmatism began emerging in the United States during the 1870s through a discussion group in Massachusetts involving Charles Peirce, William James, Chauncey Wright, Oliver Wendell Holmes Jr, and Nicolas St Johns Green, with the main view that a full understanding of a particular phenomenon cannot be achieved from a single methodological or philosophical perspective (ie, positivist or interpretivist) [35,36]. The approach was further developed by several other academics and nonacademics, including John Dewey, George Herbert Mead, and Arthur F Bentley, and is commonly associated with mixed methods research [35,36].

### Mixed Methods Research

Mixed methods research has been employed in health care research for decades [37]. Health care mixed methods research incorporates multiple methodologies, philosophies, or theoretical concepts as a means of exploring complex health-related phenomena [37].

Curran et al [28] outlined a process for developing an ICP, highlighting the importance of multidisciplinary collaboration with consumer and key stakeholder input and the integration of evidence (ie, local data and research) during the conception phase. This study was designed to incorporate these elements through the following methods to inform the development of a breech-specific ICP:

- Semistructured interviews with women who had experienced a live breech birth between 36 and 42 weeks of gestation within the past 5 years of their recruitment to the study
- An e-Delphi study with professionals having knowledge or experience of caring for women experiencing breech presentation
- A review of current clinical guidelines

Once the data have been analyzed and the ICP is formulated, the recommendations for the care for women with a breech presentation, as determined by the panel, will be compared with local [38] and international [13] breech clinical guidelines and presented in the ICP for review. All participants from the varying aspects of the study will be invited to provide feedback on the draft breech-specific ICP.

### Semistructured Interviews With Women who Have Experienced a Breech Birth

The aim of this aspect of the study was to provide insights and understanding into what women in Western Australia who are diagnosed with a breech presentation experience in order to highlight the areas of care that may need improvement.

### Women Participants

A preapproved graphic was circulated on social media sites such as Twitter and Facebook by the lead author in December 2018 as a means of generating awareness and interest in the study. The posts received 125 *shares*, which provided an effective and convenient method of *snowball* sampling. A minimum of 10 women, aged 18 years or above, who had experienced a live breech birth (ie, vaginal or by CS) between 36 and 42 weeks of gestation within the preceding 5 years were desired for this aspect of the project with the intent of continuing recruitment until data saturation was achieved (ie, no new themes emerged). Women were also required to be able to read and speak English.

### Data Collection

Interviews were conducted and audiorecorded by the lead author. The interviews took place in a location according to the women’s preferences—mainly in their homes or a neutral setting such as a local café. Women were also offered the option of a telephone or video call if it was difficult to schedule a date for a face-to-face meeting to take place.

### Data Analysis

The audio files generated by the interviews were transcribed by the lead author. Conversation not pertinent to the aims of the study were briefly summarized and filler words such as “umm,” “ahh,” and “like” were removed and were otherwise transcribed verbatim. Transcript analysis was guided by Critical Theoretical concepts to identify and describe the potential barriers, constraints, and facilitators faced by women experiencing breech presentation at the end of their pregnancy.

### Critical Theory

Critical Theory has its foundations in the Marxist tradition, which focuses on the emancipation of the working class from oppression by bourgeoisie society [39]. The tenets of this theory saw development through the early 20th century within the Frankfurt School [39,40]. Since then, the theory has seen applications mainly in the social sciences [40,41]. Critical Theories are used to examine the experiences of individuals in their social and political contexts to identify and understand power structures within their society with the intention of recommending and accomplishing change for the good of the group examined [41,42]. Michel Foucault examined the relationship between power and knowledge and how, when combined, these elements can be used in institutions such as hospitals and prisons, as a form of disciplinary control [43]. These concepts will guide the analysis of the resultant transcripts.

### The Delphi Technique

The Delphi technique was originally developed as a way for military experts to forecast the effect of advancing technologies on warfare but has since been employed across many disciplines as a way of reaching consensus regarding matters of import [44]. In medicine and midwifery, it has been utilized to determine research priorities, analyze professional characteristics and competencies, educational program development, and the expansion of midwifery practice to include a specialist skill set [45,46]. It has also been utilized to explore aspects of breech presentation and care [46-48].

### Consensus

Consensus is established through consecutive questionnaires, termed *rounds*, combined with controlled feedback. The initial round generally consists of open-ended questions, and the data obtained are used to generate statements that are distributed to the panel for evaluation in the subsequent rounds [49]. The process continues until the predetermined level of consensus is met by most statements. Levels of consensus have been reported to range from 50% to 100% in Delphi studies [49]. For this study, the predetermined level of consensus was set at ≥70% and has been used previously in breech-related Delphi studies [46,47]. An e-Delphi survey was utilized to explore and establish consensus among participants with the knowledge of or experience in caring for women diagnosed with breech presentation.

### The Delphi Process and Analysis

Questions for the first round were guided by a previous study relating to breech presentation [22] and a review of the literature. Individualized, reusable links were generated in Qualtrics and circulated to potential participants via email. Those who consented to participate were given 4-6 weeks to complete the questions. A reminder email was sent 2 weeks before the expiration of the individualized link with the option of an extension of the allotted time for completion if requested by the participant. This process was employed in each round. Each round was divided into sections, with similar topics grouped together. Each page comprised 1-4 questions including matrix questions, depending on the amount of detail being sorted, and each round spanned several pages. For each question, there was the option to provide feedback through a free text box. Before submission, participants were able to review their responses by pressing the *back* button in each section. All feedback and comments were presented to the panel in a table format along with a graphical representation of the statements that reached consensus between the second and final rounds. The statements were amalgamated where possible, added or revised based on participant feedback. On the basis of participant feedback and the quantity of data generated in the first round, the second round was divided into 3 parts to facilitate the ease of completion for participants and the ease of analysis for the research team.

### Participants

As breech care and birth are niche areas of interest compared with other health phenomena, a minimum of 10 professionals with in-depth knowledge of or experience in caring for women with breech presentation was desired for this study. The following inclusion criteria were set for the e-Delphi study: participants were required to be aged 18 years or above; have the ability to speak and read English; and have experience in supporting or caring for women during pregnancy, particularly those experiencing breech presentation. This study aims to capture a panel whose members had varying experiences of caring for women with breech presentation as experience is contextual; therefore, no predetermined years of experience was placed, a mix of convenience, purposive, and snowball sampling was used. Preapproved social media posts outlining the aim of the study and the contact details of the lead author were circulated on Twitter and Facebook groups such as the Coalition for Breech Birth and the Breech Birth Network for convenience sampling; and to the public to generate the interest of potential participants, encouraging them to make contact if they were interested in participating. All those who responded identified themselves as professionals who met the aforementioned selection criteria. This was verified throughout the processing of the data obtained from round 1. Recruitment took place between November 2018 and August 2019. The aforementioned posts were circulated numerous times. Participants were also encouraged to pass the study information to any of their colleagues who they believed might be interested in participating (ie, snowball recruitment). Recent breech literature was also reviewed as a means of identifying potential participants (ie, purposive sample). A minimum of 2 emails or direct messages through social media were sent to the participants identified through the literature.

### Clinical Guideline Review

Current guidelines on breech management and care will be purposively selected from national and professional organizations in the United States, Canada, the United Kingdom, Europe, Australia, and New Zealand for review to aid in answering the second research question (ie, What optimal pathway of care is recommended for women with a breech presentation between 36 and 42 weeks?). The authors propose using the clinical practice guideline appraisal tool International Centre for Allied Health Evidence Guideline Quality Checklist to evaluate each guideline. This tool has been validated and was deemed best suited because of its ability to efficiently and effectively review the quality of clinical practice guidelines [50]. This review will also determine whether participants’ opinions align with or diverge from the examined guidelines. Exclusions will include guidelines that have received no review within the past 5 years or have been superseded and are not available in the English language. It is proposed that the following search engines and databases will be utilized to obtain copies of the desired documents: Google, University WorldSearch, CINAHL Plus with full text, and PubMed. Reference lists of abstracted documents will be searched for any further relevant material.

### Ethical Considerations

Permission to undertake this study was granted by the University Human Research Ethics Committee (project number 19566). All participants in this study were provided with an information sheet outlining the scope of the respective studies, the contact details of the research team, and the phone number for a helpline in the event that they experienced any emotional discomfort when recalling their experiences.

Women who met the inclusion criteria and were willing to participate in a semistructured interview were required to sign a consent form before participating. Women were advised that they would be able to withdraw from the study at any time before the completion of their interview. Confidentiality of the women was maintained through the deidentification of all transcripts by using codes based on the women’s birth modes (ie, VBB1 or CS10) and the storage of sensitive documents and data in a secure location only accessible to the research team.

Participants of the e-Delphi study were required to indicate their consent to participate by answering a *yes* or *no* consent question before commencing each round. Before commencing each round, participants were advised that they would be able to withdraw from the study any time before the submission of their responses. If they withdrew or did not complete a round, they were advised that they would be excluded from future rounds.

All data were stored on the web in Qualtrics, which is password protected and only accessible to the research team, and all data were deidentified, if necessary, for consecutive rounds.

## Results

### Women’s Results

In the interviews, women described their experiences of breech presentation from their diagnosis until after birth. The data generated from the interviews with women will be examined utilizing Critical Theoretical concepts to identify and describe the potential barriers, constraints, and facilitators faced by women experiencing breech presentation near term. This aspect of the study will provide insights into what women in Western Australia experience when diagnosed with a breech presentation near term, providing context and possibly the justification for the development of the breech-specific ICP. By applying the Critical Theoretical framework, the authors hope to explore and examine the unique issues faced by women who are diagnosed with breech presentation at the end of their pregnancy to make recommendations for change to bring about a more woman-centered approach to breech care in Western Australia.

### Delphi Results

Round 1 comprised demographic and open-ended questions. The responses from this round were evaluated, coded, categorized, and amalgamated where possible to formulate statements for the following 2 rounds using the participants’ own words by the lead author. These statements were then sent to the rest of the research team along with the raw data from round 1 for review. Feedback from the research team was incorporated, and the statements were refined and rechecked before distribution to the panelists. A similar process took place during each round based on the panelist comments made in each round and feedback from the research team members.

Statements were evaluated primarily using a 5-point Likert scale, in which responses ranged from strongly agree to strongly disagree. Owing to human error during the construction of round 2 in Qualtrics, a few statements were evaluated using a 7-point Likert scale. Regardless, if the predetermined level of consensus of 70% or more of the panelists agreeing (responses ranging from somewhat agree to strongly agree) or disagreeing (responses ranging from somewhat disagree to strongly disagree) with the presented statements was reached, consensus was deemed to be met in the third and final round.

### Combining the Results

**Figure 1:**
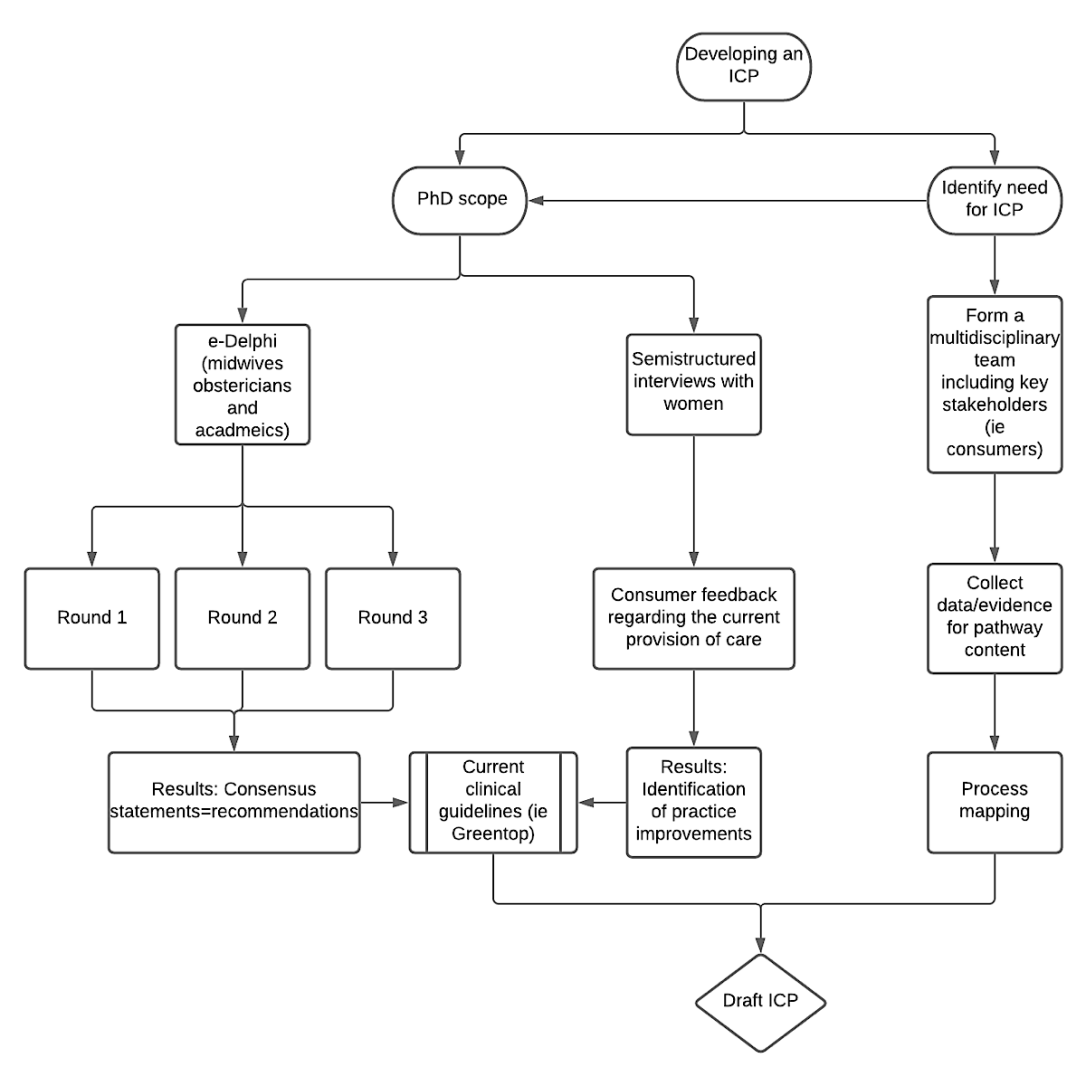
Integrated care pathway process. e-Delphi: electronic Delphi; ICP: integrated care pathway.

In-depth data analysis and review of consensus statements will be combined with an exploration of the women’s experiences of breech birth in Western Australia analyzed using Critical Theoretical concepts and current clinical guidelines to aid the formulation of an ICP for breech presentation. A process adapted from the work of Curran et al [28] was adopted to plan the aspects of a PhD project with the aim of developing an ICP (Figure 1).

### Reflexivity and Research Validity

Critical approaches emphasize the importance of researcher reflexivity [51], which acknowledges the role of the researcher as an active contributor to the construction of knowledge [52]. The lead investigator undertook an interview skill development workshop before conducting the abovementioned interviews to consolidate her previous experience. She also undertook self-reflection for the purpose of identifying preconceptions and possible biases regarding breech care and birth in order to minimize the potential effect these preconceptions and biases could have on the research.

Methods of ensuring the validity of the findings included transcript verification by participants and peer checking of the themes, concepts, and statements derived from the data from both research methods. Women who participated in the semistructured interviews were provided with a copy of their interview transcript and offered the opportunity to verify its contents based on their recollections of the interview. The resultant transcripts were also reviewed by and compared with the audio files by the coauthors to assess the accuracy of transcription. Corrections included typographical errors and 1 change in gestational age at diagnosis of breech presentation based on the woman’s recall.

Peer checking of the themes, concepts, and statements derived from each data set by the lead author was accomplished through reflective discussion and review of field notes and e-Delphi responses by the coauthors. Adaptations were made if deemed necessary based on corroborative and constructive feedback.

## Discussion

### Preliminary Agenda

Using a pragmatic, mixed methods approach to answer the original research questions has allowed the collection of qualitative and quantitative data to provide a comprehensive examination of differing aspects of breech presentation in Western Australia and various continents around the world. The results will be used to guide the formulation of practice recommendations and a breech-specific ICP that will incorporate multidisciplinary collaboration with consumer and key stakeholder input and the integration of evidence on a local and international level.

The women’s experiences explored in this study will provide insights and understanding into what some women in Western Australia have undergone throughout their breech pregnancy and birth experience and will aid in formulating recommendations to promote a more woman-focused approach to breech care and management. It is the author’s hope that a breech-specific ICP for Western Australia will facilitate this process.

An ICP for breech presentation alongside a specialty breech service has the potential to promote a more women-focused approach to breech care in Western Australia, reduce the rate of CS for breech presentation, and aid data collection for practice review and quality improvement [28].

### Benefits and Challenges

An advantage of mixed methods research includes a more comprehensive understanding of the phenomenon in focus instead of a singular point of view by incorporating qualitative and quantitative data and different theoretical perspectives [53]. This methodological approach provided flexibility in answering the complex health-related questions of this study and provided insight and depth into participants’ experiences of breech presentation [54]. A challenge for all the chosen methods was that they were time consuming for both the research team and the participants at different stages of the study [44,54]. The sample sizes for both aspects of this study were relatively small; therefore, any findings will not be generalizable.

Interviews were chosen for their ability to explore and describe issues from the perspective of the participants [55]. The interviews allowed for trust and rapport to develop between the participant and the researcher, which resulted in deeper insights into participant experiences through conversation [54]. Semistructured interviews were utilized for this study as a means of guiding the *conversation*; clarification was able to be sought immediately and the researcher had the opportunity to probe further into the aspects of the topic that were of interest to elicit more in-depth understanding [54]. However, as with all qualitative research, there was the potential for the experiences and preconceived ideas of the researcher to influence the responses and findings of the study, despite efforts to minimize these influences [54].

The advantages of the Delphi method include achieving consensus without disregarding the minority, flexibility to adapt the protocol to suit the needs of the project, cost-effectiveness if done on the web, connectivity to geographically dispersed participants, and reduction of the influence of dominant personalities on others’ responses [44,45]. However, its limitations must be acknowledged. This method is only quasi-anonymous, and there are no set guidelines regarding techniques, sample sizes, or determining consensus [44]. There is the possibility of biases occurring in Delphi studies, especially in a niche area of practice such as breech presentation, which is known to be divisive among many clinicians [2]. This may impede the achievement of a real consensus [56]. One must also acknowledge the risk of ambiguity within the questionnaire [44]. As panel members may interpret statements differently, there was also the risk of result polarization [56]. To mitigate the risk of ambiguity, a 5-point Likert scale was used; however, this is not a guarantee against the polarity of opinion [56]. Only quasi-anonymity could be offered to panel members; in a niche area such as breech, it was possible that the panel members might have known each other. However, participants were not privy to each other’s responses, and any feedback provided in consecutive rounds was deidentified if necessary.

### Conclusions

This paper describes the design of a mixed method study which will amalgamate expert opinion (consensus reached in the e-Delphi study), consumer feedback (based on the results from interviews exploring women’s experiences of breech birth and the review of exisiting breech birth guidelines [30] to guide the development of an ICP for breech presentation. A breech specific ICP has potential benefits for women and clinicians alike. These include a reduction in unnecessary intervention, streamlining care (ie, including timely referral and intervention), aiding in promoting nonbiased counseling, and improving communication between women and clinicians. This is the first of its kind in Western Australia.

## Abbreviations

CS: cesarean section
ECV: external cephalic version
ICP: integrated care pathway
VBB: vaginal breech birth
